# Efficacy of a Digital Acceptance and Commitment Therapy Intervention for the Improvement of Self-management Behaviors and Psychological Flexibility in Adults With Cardiac Disease: Protocol for a Single Case Experimental Design

**DOI:** 10.2196/33783

**Published:** 2022-04-01

**Authors:** Orla Moran, Julie Doyle, Oonagh Giggins, Louise McHugh, Evelyn Gould, Suzanne Smith, Shane Gavin, Nisanth Sojan, Gordon Boyle

**Affiliations:** 1 NetwellCASALA Dundalk Institute of Technology Dundalk Ireland; 2 School of Psychology University College Dublin Dublin Ireland; 3 Harvard Medical School Boston, MA United States

**Keywords:** cardiac disease, acceptance and commitment therapy, distress management, self-management, single case experimental design, digital health

## Abstract

**Background:**

Research indicates that the management of distress levels in those with cardiac disease is not only important for improving quality of life and functioning but also critical for condition management; adherence to treatment; and, ultimately, disease prognosis and progression. Acceptance and commitment therapy (ACT) has consistently demonstrated positive long-term outcomes across a wide array of conditions, including chronic illness. However, most empirical investigations conducted to date have also involved in-person therapy, which can be difficult to access, particularly for those dealing with the demands of chronic disease.

**Objective:**

The objective of our research is to evaluate a digital ACT intervention for improving self-management behaviors and distress levels in those with cardiac conditions.

**Methods:**

The digital ACT intervention will be delivered via a digital health self-management platform over 6 sessions. This will involve a randomized, multiple baseline, single case experimental design with approximately 3 to 15 adults with cardiac disease. The independent variable for each participant will be the pre-post intervention phase. The dependent variables will be a daily self-report measure of psychological flexibility as well as objective measures of condition self-management (eg, blood pressure readings) and engagement with the app (eg, completing guided mindfulness). One-to-one qualitative interviews will also be conducted to further examine participants’ experiences with using the intervention and what factors contribute to or impede successful outcomes.

**Results:**

Participant recruitment and data collection began in October 2021, and it is projected that the study findings will be available for dissemination by spring 2022.

**Conclusions:**

The findings will be discussed in terms of how a digital ACT intervention can best meet the needs of cardiac patients.

**International Registered Report Identifier (IRRID):**

PRR1-10.2196/33783

## Introduction

Chronic illnesses typically result in a complex set of symptoms and demanding lifestyle adjustments, with upwards of 30% of individuals with 1 or more chronic health conditions also experiencing a clinically significant mental health concern [1]. Cardiac disease in particular is linked to high levels of depression and anxiety [2-4]. Even more concerning is that depression and anxiety are associated with poor treatment adherence, poor functionality, increased hospitalization rates, and increased mortality risk across studies [5-7].

To date, cognitive behavioral therapy (CBT) is the most widely tested psychotherapeutic approach for patients with chronic conditions in the psychology literature. Although CBT has been associated with improved mood and quality of life as well as decreased symptoms of depression and anxiety [8-10], positive psychological and mood outcomes have not been consistently observed in people with chronic health conditions [11]. Many therapeutic approaches, including CBT, strive to achieve emotional and behavioral change by attempting to change or suppress “faulty” thoughts. For those with chronic conditions, this is unlikely to be feasible, particularly because condition management requires participants to engage with and report on their symptoms in an honest manner, and attempts to suppress and avoid difficult thoughts and feelings regarding symptoms can be invalidating and disempowering and may lead to or increase the avoidance of self-management behaviors [12].

In order to meet the complex needs of those with cardiac disease and to develop more effective psychotherapeutic interventions for the improvement of mental well-being, adherence, and self-management behaviors in these populations, a transdiagnostic approach (ie, universally applicable treatment regardless of a condition or diagnosis) that reduces avoidance and results in lasting outcomes is needed. One such approach may be found under the remit of contextual behavioral science (CBS).

CBS is a psychological science that provides in-depth and theoretically coherent explanations of complex human behaviors [13,14]. The clinical application of CBS is known as *acceptance and commitment therapy* (ACT) [15], and its therapeutic aim is to foster a process known as *psychological flexibility*. Hayes et al [16] reported the following definition of psychological flexibility: “the ability to contact the present moment more fully as a conscious human being, and to change or persist in behavior when doing so serves valued ends.” Distress reduction is not a goal in ACT; rather, it enables an individual to engage in a fully meaningful life despite the presence of distress by reducing ineffective behaviors and issues related to using thoughts and feelings as reasons for exhibiting or avoiding certain behaviors. ACT involves using a transdiagnostic approach, and over the last 30 years, empirical support has been observed for its use in managing a plethora of conditions, including but not limited to depression, anxiety disorder, stress, chronic illness, diabetes, addiction, chronic pain, and distress reduction in patients with cancer [16-18]. Further, even when presented in a very brief form, ACT results in positive outcomes for chronic conditions [19,20].

Despite the large body of literature demonstrating the efficacy of ACT for mental health concerns, chronic pain, and other conditions [17,18,21], there have only been a small number of investigations into ACT interventions for chronic disease conditions. However, desirable results from ACT have been observed for reported well-being, quality of life, adherence, and self-management behaviors across a range of populations, including those with diabetes, HIV, colorectal cancer, inflammatory bowel disease, obesity, and other health conditions [22-28]. To the best of our knowledge, there has only been 1 study published to date that explores the feasibility of ACT with a population of cardiac patients. Goodwin et al [29] conducted a feasibility study wherein 16 cardiac patients (12 following the drop out of 4 participants), who were mostly of lower socioeconomic status, completed 4 ACT workshops. Each workshop was conducted for 90 minutes. Large to medium improvements were observed for measures of patients’ adherence to healthy lifestyle behaviors, including weight loss, increased physical activity, and improved diet. Similarly, significant or near significant improvements were observed for psychological self-report measures of acceptance, awareness, and cognitive defusion (ie, the undermining of unhelpful thought patterns). However, due to low power and the lack of a control condition, these findings must be considered tentatively, but these preliminary results are promising [29].

A systematic review by Graham et al [30] identified a number of investigations into ACT interventions for chronic health conditions that demonstrated promising findings, including improved psychological flexibility, medication adherence, and disease self-management. Poor study quality was a considerable issue, with many studies being underpowered and lacking a control condition and the authors calling for more rigorous investigations. Most empirical investigations conducted to date however have involved in-person therapy, which typically can be expensive, time-consuming, and difficult to access, especially for those dealing with the demands of chronic disease [31]. Interventions delivered in the form of a digital application may offer a solution for these problems. Although such interventions have not been extensively investigated among patients with chronic diseases, there are promising findings emerging [27,32,33].

In addition to being more cost- and time-effective [34], web-based and digital interventions have also been shown to increase health literacy and knowledge and improve coping [35]. ACT seems particularly effective when used in combination with health education and training [23], therefore making it particularly well suited for use in the management of chronic conditions.

The aim of our study is to investigate the efficacy of a digital ACT intervention for the improvement of self-management behaviors, adherence, and psychological flexibility in a sample of participants with cardiac disease. The study will use a digital self-management platform that was developed and rigorously tested in large-scale trial with older adults with chronic health conditions, ensuring its suitability for use with such populations [36]. This technology supports participants in monitoring and reviewing symptom (eg, blood pressure, heart rate, and weight) and lifestyle parameters (eg, activity) that are relevant to their conditions and has been updated to include a newly designed app (ECME [Eastern Corridor for Medical Engineering Centre]-ACT; described further in the *Digital Platform* section). The platform also captures data on participants’ engagement with devices as well as readings, therefore providing objective measures of target behaviors in real time. Participants will also complete a daily self-report measure of psychological flexibility.

Given the transdiagnostically applicable nature of ACT, our research will examine its suitability for all levels of disease severity and progression, with the goal of developing an intervention that will (1) increase engagement with relevant preventive behaviors among people with less severe cardiac disease (eg, hypertension) and (2) provide sufficiently high treatment dosages to those with more profound levels of disease (eg, advanced heart failure), who may require a lot of distress management. Therefore, individuals who have been diagnosed with any type or stage of cardiac disease will be invited to participate in the research.

Due to both the specific nature of the population under investigation and the fact that measures must be completed on a daily basis, our study will use a randomized, multiple baseline, single case experimental design (SCED). SCEDs offer practical and effective solutions by providing high-quality evidence for the efficacy of an intervention and using a considerably smaller sample than that of a randomized controlled trial. Such a design is particularly well suited to examining health and lifestyle behaviors in real time in the context of participants’ daily lives to provide more accurate insights into how and when behavior change occurs. This can help to identify which intervention components are active (and which are not active) in producing meaningful changes.

Given the complexity of symptoms and varying levels of disease severity and prognoses among individuals with cardiac conditions, as well as the wide range of contextual factors that impact outcomes, it is important to understand these individuals’ varying needs and how they may best be met. On this basis, participants will be invited to participate in one-to-one interviews upon their completion of the program to explore in further detail the factors that contributed to or impeded successful engagement with the overall intervention and any potential issues that arose. It is predicted that self-management behaviors will improve and that psychological flexibility will increase following the completion of the digital ACT intervention.

## Methods

### Ethics Approval

Ethical approval for the study was granted by the Research Ethics Committee of our host institution. Due to the sensitive nature of the study, participants will be provided with contact details for support services and reminded of their right to withdraw at any time. They will also be reminded that they may inform the research team about any concerns or any issues.

### Design

Our research will involve a mixed methods design, which will include a nonconcurrent, randomized, multiple baseline SCED and one-to-one qualitative interviews. SCEDs treat each participant as a separate experiment in which data are collected at multiple time points for each participant and subsequently meta-analyzed across participants. This type of design is well suited to research involving niche or difficult-to-reach populations and provides advantages over group designs by examining the variability within and the experimental control of an individual’s behavior as opposed to using group means [37,38]. The independent variable for each participant will be the pre-post intervention phase, and the dependent variables will be self-report measures of psychological flexibility, objective measures of self-management, and engagement with symptom and lifestyle parameter monitoring. The intervention will be randomly staggered across participants. Prior to the intervention being administered, baseline data will be collected from each participant for 2 weeks or until a stable baseline score is established for dependent variables, at which point the intervention will be introduced. At 1 week after the introduction of the intervention (or sooner), should an effect be observed, the next participant will begin to complete the baseline data collection process, and this will continue until the intervention is introduced to all participants. Randomization periods of 5 to 7 days are recommended [39].

Consistent with the aim of CBS, that is, to examine outcomes of interest at multiple levels of evidence analysis by using various methods [40], quantitative findings will be triangulated by using qualitative interviews wherein more detailed insights into each participant’s experience with the trial will be examined.

Following the completion of the intervention, each participant will be invited to take part in a semistructured one-to-one interview, during which the following issues will be explored: the factors that facilitate and impede successful outcomes, the contextual factors that may affect one’s ability to engage with and adhere to the intervention (eg, literacy levels and time commitments), the impact of disease severity on the ability to engage with the trial (eg, pain and fatigue), and whether the intervention was delivered as intended. Negative case sampling will also be performed if any participants choose to discontinue the intervention. They will also be invited to take part in one-to-one interviews to discuss the factors that led them to discontinue the intervention.

### Recruitment

Potentially eligible participants will be invited to take part in the research via a living lab panel. The lead researcher will also contact relevant local services, including general practitioner and support services for those with cardiovascular conditions (eg, Irish Heart Foundation), and ask them to provide study details to potentially interested participants. General practitioner offices and support services will identify possible participants who meet the inclusion criteria and will provide them with the participant information leaflet, which contains details for contacting the lead researcher about participation.

If an individual wishes to participate, the lead researcher will phone them to discuss the study and arrange to obtain written informed consent. The inclusion criteria will include being over 40 years of age and having a diagnosis of cardiac disease, and the exclusion criteria will include symptoms of psychosis or suicidality, severe cognitive impairment or learning difficulties, or a below conversational level of English. For a multiple baseline SCED, samples of 3 or more participants are recommended [41]; therefore, in order to account for missingness and participant attrition, a slightly larger sample of approximately 10 to 15 participants who meet the criteria will be recruited.

### Procedure

**Figure 1:**
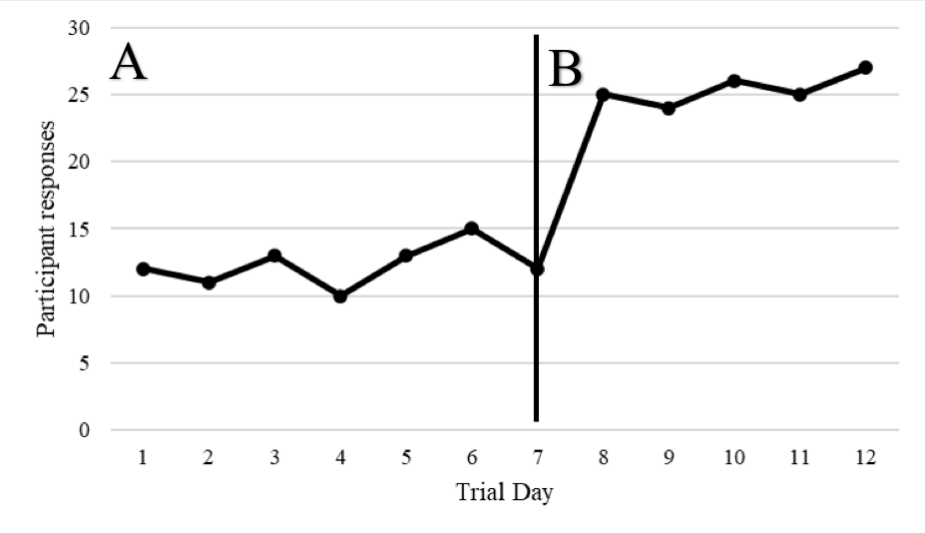
Graphical display of hypothetical A-B single case experimental design data [41].

Upon agreeing to participate in the study and once informed consent is obtained, participants will receive an Apple iPad (8th Gen, 10.2-inch, Wi-Fi, 32GB; Apple Inc) with the ECME-ACT app installed as well as digital devices for monitoring symptoms and lifestyle parameters of relevance to their cardiovascular condition. Participants will either meet the researcher in their homes or in the research center (depending on their preferences and availability) wherein they will receive the devices and be verbally instructed on how to use them in addition to being provided with the relevant user manuals. Upon receiving the app (but prior to the ACT intervention being administered), baseline data for digital self-management behaviors and the self-report measure of psychological flexibility (see *Measures* section) will be collected for each participant over a 2 week period or until a stable baseline score is established. A multiple baseline SCED will be used, wherein the intervention will be staggered across participants (Figure 1).

Participants will complete daily measures and homework by using the digital app, and the ACT intervention will be delivered by 2 ACT therapists via Zoom (Zoom Video Communications Inc) in once-weekly sessions over the course of 6 sessions at a particular time or on a day that suits each participant.

### Measures

The Brief Acceptance Measure [42] is a 3-item self-report measure of psychological flexibility that was designed specifically for use in SCEDs. Participants will complete this daily within the app and will be sent a reminder to do so via a text message (sent by the primary researcher).

Symptom and lifestyle data and engagement with monitoring will be logged via the digital app for relevant self-management behaviors (eg, monitoring blood pressure and inputting weight). Participants will also receive a daily text message reminder to take readings. Engagement with the digital app will also be logged to ascertain the number of times that the participants use the app and its various features.

One-to-one interviews will examine participants’ experiences with the intervention and explore the following:

- How are quality of life and day-to-day functioning following the completion of the intervention?
- What factors facilitated or impeded the ability to engage with the intervention?
- What changes to the intervention, if any, would participants make?
- If applicable, what factors lead the participant to discontinue their use of the digital intervention?

### ACT Intervention

The intervention used in the study will be based on the *Acceptance and Commitment Therapy (ACT) Training Manual for Stress Reduction in Patients with Inflammatory Bowel Disease (IBD)* protocol [43], which will be adapted for use with cardiac conditions as opposed to inflammatory bowel disease. This protocol outlines a 6-session ACT intervention for populations with chronic illness with once-weekly sessions (approximately 1 hour). The intervention in this study will be delivered via Zoom by a peer-reviewed ACT trainer and expert and the lead researcher. Participants will also be provided with psychoeducation and mindfulness exercises (homework) via the digital app to help them implement and practice the strategies that they have learned throughout their day-to-day lives.

### Treatment Fidelity

Sessions will be recorded and assessed by the lead researcher to ensure treatment fidelity and consistency.

### Digital Platform

The ECME-ACT platform consists of the following components.

#### ECME-ACT App

**Figure 2:**
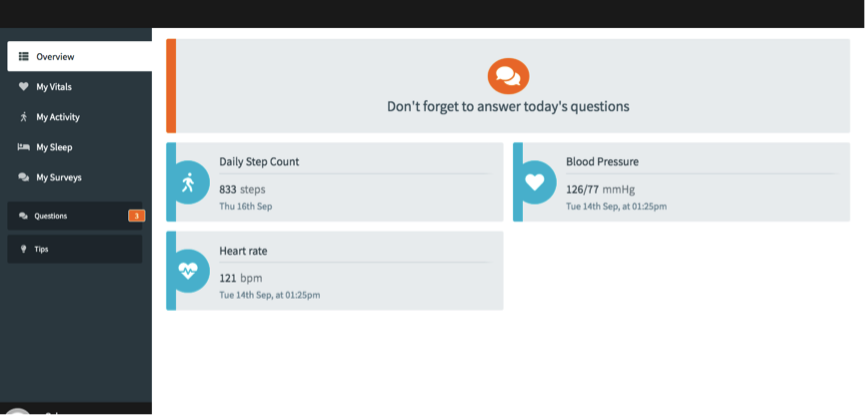
ECME-ACT app dashboard. ACT: acceptance and commitment therapy; ECME: Eastern Corridor for Medical Engineering Centre.

**Figure 3:**
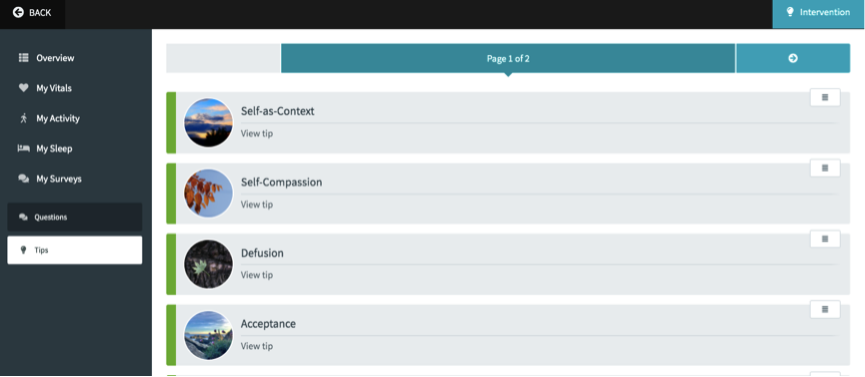
ECME-ACT app: tips and psychoeducation. ACT: acceptance and commitment therapy; ECME: Eastern Corridor for Medical Engineering Centre.

**Figure 4:**
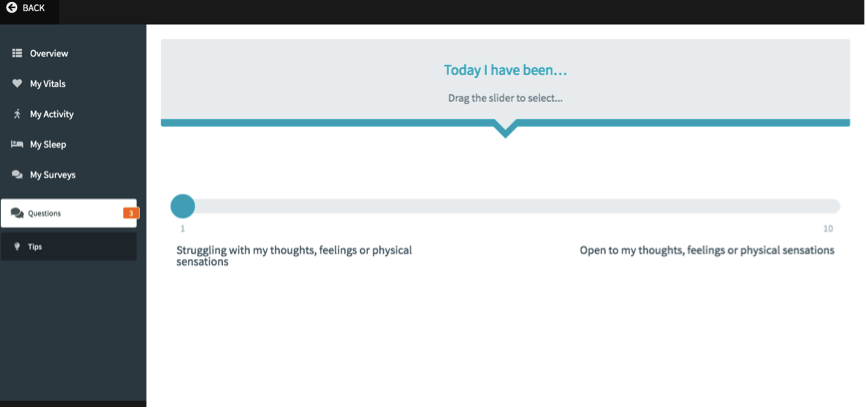
ECME-ACT app: daily self-report questions (Brief Acceptance Measure [43]). ACT: acceptance and commitment therapy; ECME: Eastern Corridor for Medical Engineering Centre.

The ECME-ACT app is the responsive web-based app on which participants will engage with data from digital health devices, including blood pressure, heart rate, weight, activity, and sleep (Figure 2), and features related to the digital ACT intervention (specifically, guided mindfulness audio recordings, psychoeducation and tips [Figure 3], and daily self-report measures [Figure 4]). The design of the digital app is based on findings from previous research, including interviews and co-design sessions, involving older adults with cardiac conditions [36,44]. Participants will be provisioned with an iPad for the duration of their participation in the study, through which they will engage with the ECME-ACT app and participate in the one-to-one therapeutic sessions conducted via Zoom.

#### Context-Aware Broker and Inference Engine (Plus)

CABIE+ (Context-Aware Broker and Inference Engine [Plus]) is the data collection and aggregation system that will be used to organize and store the data acquired from the ECME-ACT app and integrated digital devices.

#### Subject Information Management System

**Figure 5:**
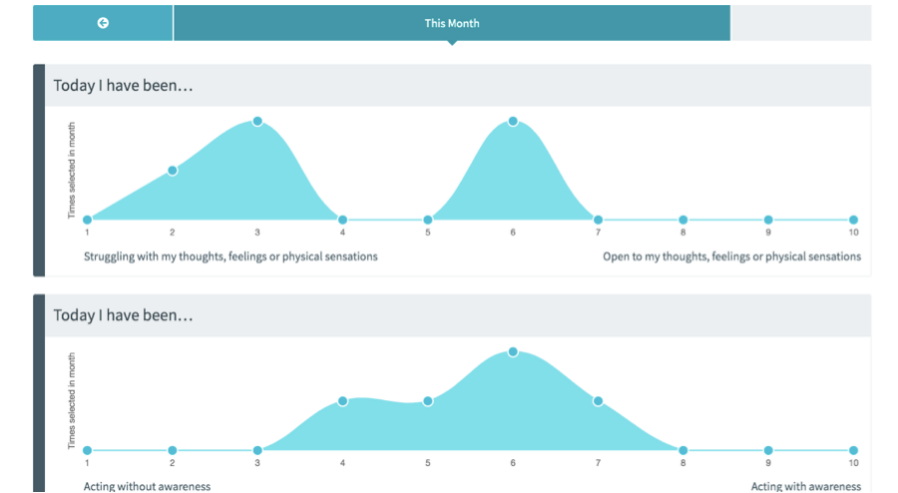
SIMS interface showing the inspection of daily self-report question data. SIMS: Subject Information Management System.

SIMS (Subject Information Management System) is the information management system that will be used to allow the research team to view, analyze, and interpret the data collected from the app and the devices in close to real time for individual participants, including engagement data (Figure 5; further details are provided by Doyle et al [36]).

### Digital Devices

Two off-the-shelf consumer devices—the Withings Smart Watch Activity Tracker (Withings) and the Withings BPM Connect (Withings)—are integrated with the digital platform. They will be used to collect health and well-being data over the course of the study. The Withings Smart Watch is a high-end smart device designed to monitor activity and sleep. The Withings BPM Connect has been clinically validated in terms of its ability to measure blood pressure and heart rate.

### Analytic Strategy

#### Quantitative Analysis

Although SCED investigations typically involve visual data analyses [45], visual data inspection has been found to have low rates of interrater reliability [46-48]. Therefore, data will be analyzed quantitatively as well as visually in the study. Data will be analyzed quantitatively by using the SCED R package [49]. This R package was designed specifically for use in A-B SCEDs, and it allows for robust analyses, the plotting and meta-analysis of A-B SCED data, the use of exact tests, and the calculation and meta-analysis of robust effect sizes.

Raw data will also be visually plotted, allowing for the visual assessment of baseline trends, between-phase differences, and variability across each phase. Modified Brinley plots will be used to visually display data. Modified Brinley plots offer a means of conveniently identifying and interpreting changes at an idiographic level [50,51]. Modified Brinley plots also allow for the inclusion of cutoff points based on a measure’s reliable change index, meaning that clinically meaningful idiographic patterns can be quickly observed and interpreted [51].

#### Qualitative Analysis

A thematic analysis [52] will be performed to analyze one-to-one interviews. The analysis will be primarily deductive in nature; the researchers will use a top-down approach guided by the research questions to generate themes and patterns within the data. Following the completion of the initial coding by the primary researcher, all study authors will be consulted to discuss, recode, and categorize codes into themes until agreement is reached on all themes and subthemes. All interviews will be coded in full by 2 separate coders and then matched to assess interrater agreement by using the Cohen κ.

## Results

Participant recruitment and data collection began in October 2021. The dissemination of study results in peer-reviewed journals is expected in spring 2022.

## Discussion

Our study will examine the efficacy of a 6-session digital ACT intervention for improving the outcomes—self-management behaviors, treatment adherence, and psychological flexibility—of a sample of cardiac patients. By using previously validated self-management technology [36], participants’ engagement with devices, as well as readings, will be captured in real time, therefore providing objective measures of target behaviors. It is predicted that improvements in self-management and treatment adherence, as well as increased psychological flexibility, will be observed following the digital ACT intervention. Should this digital intervention be found to be effective, it may address the need for transdiagnostically applicable, accessible, and wide-reaching treatment for improving the management of cardiac disease. Our findings will be of interest to health care professionals, psychologists, researchers, and policy makers.

## Abbreviations

ACT: acceptance and commitment therapy
CABIE+: Context-Aware Broker and Inference Engine (Plus)
CBS: contextual behavioral science
CBT: cognitive behavioral therapy
ECME: Eastern Corridor for Medical Engineering Centre
SCED: single case experimental design
SIMS: Subject Information Management System
